# Managing Protrusio Acetabuli With a Direct Anterior Approach Total Hip Replacement

**DOI:** 10.7759/cureus.14048

**Published:** 2021-03-22

**Authors:** Andrew Yun, Marilena Qutami, Eric Carles

**Affiliations:** 1 Orthopedic Surgery, Center for Hip and Knee Replacement, Providence Saint John's Health Center, Santa Monica, USA

**Keywords:** protrusio acetabuli, total hip replacement, direct anterior approach, bone graft, fluoroscopy

## Abstract

Introduction

The deformities of protrusio acetabuli (PA) present unique reconstructive challenges. An incarcerated femoral head, medialized center of rotation, deficient bone stock, and associated leg length discrepancy add significant technical complexity to total hip arthroplasty (THA).

Methods

We retrospectively reviewed 23 THAs in 21 patients with PA who underwent direct anterior (DA) approach THA with intraoperative fluoroscopy. All acetabular defects were reconstructed with morcellized femoral head autograft using Bone Mill (Stryker Corporation, Kalamazoo, MI).

Results

The mean AK distance preoperatively was 8 mm (range: 1-16). Postoperatively, the degree of protrusio improved in all cases, and the mean AK distance decreased to 0 mm. All bone grafts consolidated, and no cups loosened or were revised at a mean of 5.3 years of follow-up. The mean Hip Disability and Osteoarthritis Outcome Score, Joint Replacement (HOOS, JR) at follow-up was 91.

Conclusions

These data suggest that the DA approach with intraoperative fluoroscopy may be a reasonable technique in the surgical management of this challenging population.

## Introduction

Protrusio acetabuli (PA) was first described by Otto in 1824 as the intrapelvic protrusion of the acetabulum into the lesser pelvis [1]. Almost 200 years ago, the clinical findings of cartilage loss with arthritis were noted, and the pathophysiology of upward and medial migration from femoral head pressure was surmised. While the etiology of primary PA is still unclear today, secondary PA is attributable to inflammatory, genetic, and traumatic causes.

The management of clinically significant PA remains limited. Attempts to halt progression with triradiate fusion are at best limited to juvenile patients [2]. Valgus intertrochanteric osteotomy to reduce head-neck impingement fares poorly in those over 40 years of age [3]. Total hip arthroplasty (THA), therefore, may be the most reasonable of surgical options despite the increased technical complexity of acetabular reconstruction.

The challenges of PA in THA are well-documented. Anatomically, poor bone stock, a thinned medial wall, and osteoporosis undermine structural stability. Surgically, an incarcerated head, limited range of motion (ROM) for exposure and dislocation, and distorted anatomy intensify the intraoperative struggles. Reconstructively, limited medial acetabular support, an eccentric center of rotation (COR), and a relative decrease in hip length and offset potentially compromise implant position and fixation.

To address these unique challenges of PA in complex primary THA, we explored the role of a direct anterior (DA) approach with adjunctive intraoperative fluoroscopic guidance. In recognizing the intraoperative structural challenges, we theorized that this modified technique with adjunctive imaging would guide more accurate reconstructive choices to improve implant stability and to decrease postoperative complications. Therefore, the purpose of this study is to examine the technical details, clinical outcomes, radiographic metrics, and failures of a DA approach with fluoroscopy for PA.

## Materials and methods

**Figure 1:**
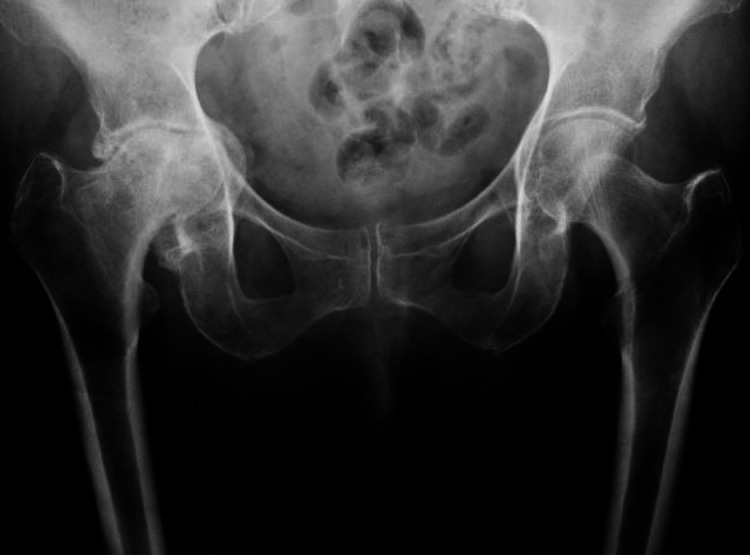
Anteroposterior radiograph of the pelvis showing characteristic protrusio acetabuli deformities. Note medial displacement of right femoral head past Kohler’s line, acetabular bone stock deficiency, incarcerated femoral head, and reduced hip offset and length.

We retrospectively reviewed a total of 23 consecutive THAs performed between 2008 and 2019 in 21 patients with PA (Figure 1). Surgery was performed by a single surgeon at a single institution with a minimum of one-year follow-up. In all patients, a direct anterior (DA) approach THA with intraoperative fluoroscopy was performed. Charts were reviewed for indications, medical history, and mode of treatment failure. Hospital records were reviewed for complications, reoperations, and rehospitalization. Outcomes measured were patient-reported HOOS, JR (Hip Disability and Osteoarthritis Outcome Score, Joint Replacement) validated scores, revision, and death [4]. The study was approved by the Institutional Review Board.

Surgical technique

The patient was placed on the Hana® table in the standard fashion. A DA approach was performed as described by Matta et al. [5]. A standard dissection was taken to the level of the hip. After capsulotomy, the hip joint was distracted with mild traction, and the ligamentum teres was divided. Proximal control of the femoral head was gained with a corkscrew, and a gentle attempt was made to dislocate the head anteriorly with external rotation. If the head was incarcerated, an in-situ neck osteotomy was performed with fluoroscopic guidance. The head was then removed wholly or in sections from the acetabulum. This bone was then morcellized with a Bone Mill (Stryker Corporation, Kalamazoo, MI) for eventual autografting.

Attention was then turned to acetabular preparation. Unlike primary THA, the acetabulum was reamed to establish primarily a peripheral rim fit. In bypassing smaller reamer sizes typically used to expose the medial wall, the larger reamers were aimed superomedially to shape the acetabulum’s periphery. The bone was prepared until a wide, circumferential stripe of subchondral bone was exposed at the periphery. The gross appearance resembled a dual hemisphere with a wider outer circumference in which the cup would anchor and an inner narrower hemisphere that the autograft would fill. Fluoroscopic evaluation of the reamer position confirmed a lateral displacement of the planned COR from the original COR. The medial acetabular bone was then prepared with curettes to remove overlying cartilage and soft tissue. Morcellized autograft from the femoral head was then impacted and pressurized with reverse reaming until the creation of a uniform, concave hemisphere matching the diameter of the outer periphery. The real implant was then impacted and evaluated with fluoroscopic guidance for proper abduction and anteversion as well as for sufficient graft-implant contact and lateralization of the COR. Screws were used if necessary to augment stability.

**Figure 2:**
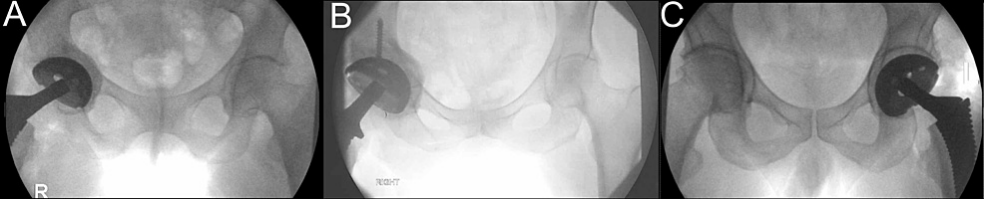
Three examples of intraoperative fluoroscopy for real-time anteroposterior imaging of the pelvis. (A) Right hip with medial bone restoration. (B) Right hip cup placement requiring adjunctive screw fixation for stability. (C) Left hip with trials in place for measurement of hip length and offset.

On the femoral side, the affected hip presented with a decreased hip length and offset. Preoperative and intraoperative templating was used to estimate the amount of combined correction from the acetabulum and the head-neck-stem construct. With the trials in place, intraoperative fluoroscopy was used to confirm the symmetry of hip length and offset compared to the contralateral side or to guide any adjustments if the trials revealed continued inequality (Figure 2). The trials were tested clinically for stability, range of motion, and impingement before the final implants were placed.

Radiographic measurement

**Figure 3:**
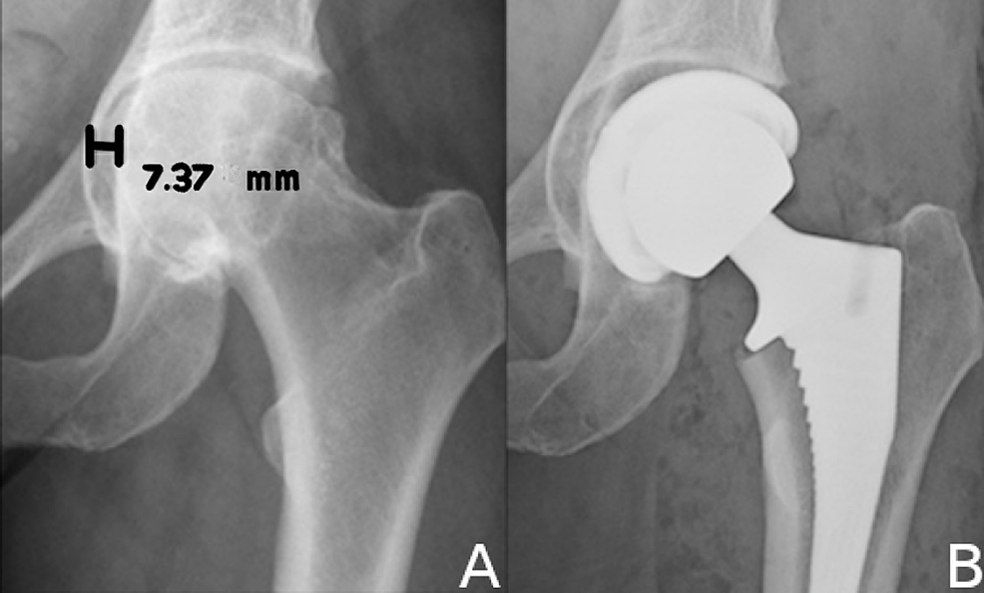
Measurement of radiographic outcomes after acetabular reconstruction. (A) Anteroposterior (AP) radiograph close-up of left hip with moderate protrusio of 7.37 mm medialization. (B) AP radiograph close-up of left hip after medial autografting and corrected center of rotation. Note the medial edge of the acetabular component is now lateral to Kohler’s line.

Preoperatively, the presence and degree of protrusio were measured on an anteroposterior (AP) pelvic radiograph. PA was noted on those hips with a center edge angle (CEA) greater than 50 degrees as described by Steel et al. [2]. Additionally, the degree of PA was measured by the horizontal displacement of the medial acetabular border that passes beyond Kohler's line (AK distance). Kohler’s line, also known as the ilioischial line, represents the radiographic landmark of the quadrilateral plate. Protrusio is mild between 1 and 5 mm, moderate between 6 and 15 mm, and severe beyond 16 mm. Postoperatively, the AK distance was measured as the distance between the medial edge of the acetabular component and Kohler’s line (Figure 3). Preoperative leg length inequality was compared to postoperative measurements. Leg length was determined by the vertical distance from a specified point on the lesser trochanter to a horizontal line drawn across the bottom of each radiographic teardrop. Inequality was calculated by the difference in hip lengths between the index side and the contralateral side.

Postoperative radiographs were evaluated for cup orientation, amount of lengthening, and residual leg length inequality. Anteversion was calculated using the opening of the ellipse as calculated by the Radlink software (Radlink, El Segundo, CA). The acetabular bone was evaluated for ingrowth and graft consolidation. The implants were evaluated for loosening, migration, and radiolucent lines as previously well-described.

## Results

Patients included 19 women and two men. Two of the women underwent staged bilateral DA THA. The average age of patients at time of index surgery was 68 years (range: 43-81). No patient had undergone prior surgical correction. Five patients (22%) had rheumatoid arthritis, and the remaining 18 patients (78%) were diagnosed with idiopathic PA.

At the time of surgery, the average estimated blood loss (EBL) was 182 mL (range: 50-400). Cementless acetabular cups and femoral stems were used in all cases. There were no known intraoperative complications. One patient required a transfusion during hospitalization. The average length of stay was three days (range: 1-20). Nineteen patients were discharged home, and four patients were discharged to a skilled nursing facility. Descriptive statistics, demographics, and clinical outcomes are shown in Tables 1, 2, respectively.

**Table 1:** Descriptive statistics including demographic information and diagnosis.

Measurements	
No. of cases	23
No. of patients	21
Age (years)	
Mean/SD	68 ± 9
Range	43–81
Gender	
Male	2 (10%)
Female	19 (90%)
Laterality	
Right	11 (48%)
Left	12 (52%)
Preoperative diagnosis	
Rheumatoid arthritis	5 (22%)
Idiopathic protrusio acetabuli	18 (78%)

**Table 2:** Clinical outcomes. HOOS, JR: Hip Disability and Osteoarthritis Outcome Score, Joint Replacement.

Measurements	
Estimated blood loss at time of surgery (mL)	
Mean/SD	182 ± 104.2
Range	50–400
Length of stay (days)	
Mean/SD	3 ± 3.8
Range	1–20
No. of patients discharged to	
Home	19 (83%)
Skilled nursing facility	4 (17%)
Time to follow-up (years)	
Mean	5.3 ± 3.2
Range	1–12
HOOS, JR score (percent)	
Mean	91 ± 10.4
Range	70–100

Radiographic outcomes

The mean CEA preoperatively was 65 degrees (range: 54-82). Preoperatively, the mean AK distance was 8 mm (range: 1-16). There were seven patients with mild PA, 13 with moderate PA, and three with severe PA. Postoperatively, the degree of protrusio improved in all cases, and the mean AK distance decreased to 0 mm. Ten hips had residual mild protrusio between 1 and 4 mm. In the remaining 13 hips, the medial edge of the acetabular shell was moved lateral to Kohler’s line. For the acetabular implant, the mean abduction angle was 45 degrees (range: 41-48), and the mean anteversion angle was 18 degrees (range: 13-24). For limb length inequality, the average amount of preoperative shortening was 4 mm (range: 0-11). The average amount of postoperative limb length inequality was 2 mm (range: 0-4). The average amount of operative limb lengthening was 3 mm (range: 0-10). At follow-up, all hips showed graft consolidation and no evidence of acetabular shell loosening. Descriptive statistics are given in Table 3.

**Table 3:** Pre- and postoperative radiographic results. PA: Protrusio acetabuli.

Measurements			
Preoperative leg length discrepancy (mm)		Postoperative leg length discrepancy (mm)	
Mean/SD	4 ± 3.3	Mean/SD	2 ± 1.5
Range	0–11	Range	0–4
Preoperative AK distance (mm)		Postoperative AK distance (mm)	
Mean/SD	8 ± 4.5	Mean/SD	0 ± 2.2
Range	1–16	Range	-4–3
Preoperative degree of PA (No. of cases)		Postoperative degree of PA (No. of cases)	
Mild	7 (30%)	Mild	10 (43%)
Moderate	13 (57%)	Moderate	0 (0%)
Severe	3 (13%)	Severe	0 (0%)
Preoperative center edge angle (degrees)		Acetabular cup abduction angle (degrees)	
Mean/SD	65	Mean/SD	45 ± 1.9
Range	54–82	Range	41–48
		Acetabular cup anteversion angle (degrees)	
		Mean/SD	18 ± 3.1
		Range	13–24

Functional outcomes

The mean follow-up was 5.3 years (range: 1-12). At final follow-up, the average HOOS, JR was 91 points (range: 70-100). A preoperative HOOS, JR score was not available for comparison.

Complications

One patient was revised 14 weeks after index surgery for stem subsidence. One month after an uneventful hospital course, the patient was noted to have a limp, 1 cm of stem subsidence, and mild thigh pain. Given her fragile state of health and the well-documented possibility of ingrowth after subsidence, she was treated conservatively for eight weeks. Her stem restabilized and her pain fully resolved, but she could not tolerate the leg length discrepancy. She underwent revision of her stem through a posterior approach and is doing well in follow-up.

Another patient with Meckel’s diverticulum required 20 days of inpatient care for small bowel obstruction and acute pyelonephritis. Three months later, she was walking comfortably without assistive devices indoors and a walker for balance outdoors. In both of these patients, the autograft and acetabular shell healed without incident.

## Discussion

Achieving uniformly successful outcomes in primary THA is difficult enough, thus the added complexity of stiffness and deformity of PA warrant ongoing and further consideration. Distorted landmarks create surgical uncertainty, and an increased CEA is concerning for potential implant and bone impingement. Among the many challenges of PA are cup lateralization and fixation [6], reconstruction of medial bone support with grafting [7-9], and equalization of planned length and offset to minimize impingement. Historic methods heretofore have focused on the posterior or lateral approaches without adjunctive imaging. To our knowledge, there are no known articles related to the use of the DA approach in treating patients with PA. Therefore, this is a rare description of a consecutive series of patients with PA treated with DA approach total hip arthroplasty (THA).

**Figure 4:**
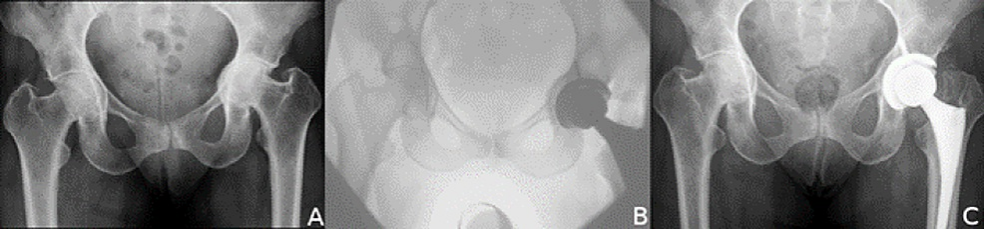
Protrusio acetabuli managed with the direct anterior approach and intraoperative fluoroscopy. (A) Preoperative anteroposterior (AP) pelvic radiograph with typical protrusio deformities. (B) AP intraoperative image to assess medial graft, cup orientation, and leg length. (C) Six-year follow-up AP pelvic radiograph with consolidation of bone graft, no radiolucent lines, restoration of offset, and correction of leg length discrepancy.

Preparation of the cup in PA has evolved and may be reflective of changes in implant technology and technique. Cemented cups were used in the earliest reported series, and studies showed survival rates of only 76% to 86% [10,11]. Modern techniques are now directed at a peripheral rim fit to secure fixation and medial grafting to lateralize the COR; the rim of subchondral bone provides circumferential fixation, and the autograft provides polar support [7,8]. With adjunctive fluoroscopy, intraoperative images provide clear information about the extent of protrusio correction, which is reflected by the change in the AK distance. In our study, the surgical technique successfully lateralized the COR and decreased the mean preoperative AK distance from 8 to 0 mm with no acetabular failures reported. In addition to confirming the efficacy of rim fit fixation in this series, we also found that placing the cup under fluoroscopic guidance gave a visual confirmation of accurate implant positioning with a mean of 45 degrees abduction and 18 degrees of anteversion (Figure 4).

Additionally, managing the deficient medial wall is essential for stabilizing the acetabular shell and restoring bone stock for future demands. The need for supplementary bone graft has been described in prior studies. Krushell et al. added synthetic graft in three of 29 cases, and Zhen et al. needed adjunctive graft from the iliac crest through a separate incision in eight of 20 hips [9,7]. In contrast, we found morcellization of the femoral head with a commercial bone mill to be efficient and to provide more than enough autograft to restore the COR. Although some have suggested that commercial bone mills create graft particles that are too small for ingrowth [8], we did not appreciate any poor outcomes related to ingrowth or consolidation with this method.

Furthermore, the DA approach may mitigate the incidence of dislocation in these high-risk patients. We speculate that historic rates of instability may be associated with a combination of the surgical approach and the preoperative deformity that requires greater soft tissue dissection. A small series of Marfan’s patients with PA by Thakkar et al. reported a dislocation rate of 10.9% after a posterior approach [12]. In contrast, Mullaji et al. noted no dislocations using an anterolateral approach to protect against posterior instability [8]. Similarly, our series had no dislocations at the time of follow-up. We speculate that preserving the posterior capsule and tissues may be protective in this at-risk population. We further speculate that accurate restoration of hip length and the COR decreased the risk of impingement-related dislocations.

There are several limitations of this study. Primarily, it is a single surgeon’s small retrospective series. Larger numbers of patients may reveal a complication profile similar to that of other series. Another major limitation is the lack of a control group for comparison. A comparative cohort of similar patients with PA treated with the DA approach without fluoroscopy would clarify intraoperative imaging as the independent variable rather than the approach. Conversely, a cohort of patients treated with a posterior approach and intraoperative imaging could potentially report favorable findings similar to ours. The purpose of this analysis, however, was not to argue for a superior approach or technique for PA patients; rather, it was to examine the role of an anterior approach with intraoperative imaging to decrease the risk of known complications in a consistently challenging population. Another concern is that a minimum two-year follow-up is more common, but we chose a minimum one-year follow-up for analysis because our primary endpoints were perioperative complications and implant position.

## Conclusions

PA remains an uncommon indication for primary THA. Thus, the issues of head incarceration, medial wall deficiency, and peripheral rim fixation are not only challenging but also unfamiliar or underrecognized. However, modern techniques of cementless fixation at the periphery and autografting have improved upon historical results. As noted in this series, the underlying protrusio can be corrected biomechanically, and patient-reported outcomes can be favorable. An anterior approach may also decrease the significant risks of dislocation by avoiding posterior dissection. Ongoing advances in adjunctive intraoperative imaging with fluoroscopy may further improve the accuracy of implant placement and lateralization of the COR as well as reduce the stress of intraoperative uncertainty. Finally, in candidly presenting our complications, it is also a reminder that the surgical management of these patients is not without risk.
